# Outcomes of primary membranous nephropathy refractory to immunosuppressants

**DOI:** 10.1093/ckj/sfae113

**Published:** 2024-04-15

**Authors:** Saurabh Nayak, Prabhat Chauhan, Vivekanand Jha, Raja Ramachandran, Bhadran Bose

**Affiliations:** Department of Nephrology, All India Institute of Medical Sciences, Bathinda, India; Department of Nephrology, Indian Naval Hospital Ship Asvini, Mumbai, India; George Institute for Global Health, UNSW, New Delhi, India; Prasanna School of Public Health, Manipal Academy of Higher Education, Manipal, India; School of Public Health, Imperial College, London, UK; Department of Nephrology, PGIMER, Chandigarh, India; Department of Nephrology, Nepean Hospital, Kingswood, Australia

To the Editor,

Primary membranous nephropathy (PMN) is one of the most common causes of adult-onset nephrotic syndrome. The current Kidney Disease Improving Global Outcomes (KDIGO) guidelines recommend immunosuppressive therapy for PMN patients with at least one risk factor for disease progression [1]. Treatment modalities include cyclophosphamide plus steroids, rituximab, or calcineurin inhibitors (CNIs). Unfortunately, 20%, 40%, and 50% of the patients treated with cyclophosphamide plus steroids, rituximab, and CNIs continue to have persistent nephrotic syndrome, respectively [2–4]. Hence, a significant proportion of them need rescue therapy. However, the choice of rescue treatment depends on the initial regimen, the physician or patient's preference, and availability. There are no head-to-head comparisons to guide nephrologists in choosing rescue therapy. Henceforth, we undertook this systematic review to identify the best treatment strategy for managing patients with refractory membranous nephropathy.

We conducted a comprehensive electronic search (Supplementary Table S1), including all published studies that reported treatment of refractory membranous nephropathy. The primary outcome was complete remission. Secondary outcomes included partial remission and progression to kidney failure. Findings from the included articles are presented and analysed using a tabular method and narrative synthesis.

Only 18 observational studies with 125 patients were included in this review (Supplementary References 1–18). Supplementary Table S2 summarizes all the included studies, and Supplementary Table S3 describes the baseline characteristics. There were eight rescue therapies analysed in this review, which included rituximab, adrenocorticotrophic hormone, Obinutuzumab, CNI's plus steroids, cyclophosphamide plus steroids, mycophenolate mofetil, bortezomib plus dexamethasone, and rituximab plus daratumumab. Supplementary Table S4 summarizes all the treatments received before rescue therapy.

The complete remission rate was 60% with obinutuzumab, 26% with rituximab, 27% with cyclophosphamide, 20% with adrenocorticotrophic hormone, and 23% with a calcineurin inhibitor. The partial remission rate was 30% with Obinutuzumab, 30.4% with rituximab, 45.5% with cyclophosphamide, 41% with adrenocorticotrophic hormone, and 46% with a calcineurin inhibitor. There was no report of progression to kidney failure.

Despite the currently available treatment options, 15 to 45% of patients are refractory to their first line of treatment. The response is correlated by multiple factors, such as the degree of proteinuria, PLA2R titre, choice of initial immunosuppressive agents, and their toxicity/tolerability [5]. Management of patients with refractory membranous nephropathy is challenging. The latest KDIGO guidelines have given recommendations based on expert opinions on managing patients refractory to CNIs, a combination of cyclophosphamide plus steroids, and rituximab [1]. However, there are no recommendations on how to manage patients resistant to two or all three regimens. Newer agents such as ACTH, obinutuzumab, and bortezomib have been reported to manage patients with membranous nephropathy, but their efficacy is unknown. The definition of refractory disease is tricky, especially in patients who are anti-PLA2R antibody negative. How long to wait after treatment before labelling the patient refractory to a particular treatment strategy is yet to be determined. The refractory disease may additionally be assessed by the persistence of anti-PLA2R antibodies at high or unchanged levels or beyond a specific threshold level in patients with anti-PLA2R antibodies positive membranous nephropathy. In patients with anti-PLA2R antibody-negative disease, the persistence of nephrotic syndrome should be used to define refractoriness.

To our knowledge, this is the only systematic review which has reviewed the treatment for refractory membranous nephropathy, and it has identified multiple treatment options for patients with refractory membranous nephropathy. However, there are some limitations to our review. All the included studies are case series or uncontrolled studies, which raises the possibility of reporting bias. Other limitations include the fact that there is no clear definition of refractory membranous nephropathy, the definition of remission is very heterogeneous, the baseline clinical characteristics of the patients are very heterogeneous, and PLA2R antibody titres have not been reported in all the studies.

Among multiple available agents, rituximab, ACTH, and obinutuzumab show promising results, but the remission rates are variable, with no clear winner. The agents should be chosen based on the individual patient's clinical characteristics and the side effect profile of the agents. We need further controlled studies investigating these agents. However, this review has systematically analysed each treatment option and its efficacy. This helps clinicians choose an appropriate agent for patients with refractory membranous nephropathy.

## Supplementary Material

sfae113_Supplemental_File
