# Supplementary material for: Outcomes of primary membranous nephropathy refractory to immunosuppressants
Source: Clin Kidney J. 2024 Apr 15;17(5):sfae113. doi: 10.1093/ckj/sfae113 (PMC11079664; doi:10.1093/ckj/sfae113)
Supplement: sfae113_Supplemental_File [file sfae113_supplemental_file.docx]

**Electronic Supplementary Material**

**Table S1.** Search strategy.

| **Database** | **Search strategy** |
| --- | --- |
| PubMed, Embase, Cochrane Library | Refractory [tiab] OR Resistant [tiab] OR Treatment [tiab] OR Therapy [tiab] AND PLA2R-associated OR PLA2R-related OR PLA2R-positive OR Idiopathic OR Primary AND Membranous Nephropathy |

**Table S2.** Summary of the included studies

| Patient  No. | Study | Eligible | Anti-PLA2R | Baseline IS regimen | Intervention | Clinical outcome | Follow up |
| --- | --- | --- | --- | --- | --- | --- | --- |
| P1-5 | Desanto NG et al; *Am J Nephrol*, 1987 [1] | 05 | Not reported | All 5 patients were treated with chlorambucil plus steroids | cyclosporine plus steroids | 4 PR | 3-6 months |
| P6-13 | Miller G et al; *Am J Kidney Dis* 2000 [2] | 08 | Not reported | Five patients received cyclophosphamide plus steroids and 3 patients received cyclosporine plus steroids as first line therapy.  Three patients received chlorambucil plus steroids and 1 patient received cyclosporine plus steroids as second line therapy  Three patients received cyclosporine as third line therapy | mycophenolate mofetil | 1 PR, 7 NR | 6 months |
| P14-15 | Arikan H et al; *J Nephrol* 2008 [3] | 02 | Not reported | Both patients were treated with cyclophosphamide plus steroids as first line therapy  One patient received cyclosporine plus steroids as second line & mycophenolate mofetil as third line therapy | tacrolimus plus steroids | Both PR | 6-24 months |
| P16-25 | Bomback AS et al; *Drug Des Devel Ther* 2011 [4] | 10 | Not reported | Six patients received cyclophosphamide plus steroids, 3 patients received mycophenolate mofetil and 1 patient received tacrolimus plus steroids as first line therapy  Six patients received tacrolimus plus steroids as second line therapy  Two patients received mycophenolate mofetil as third line therapy | ACTH | 3 CR, 6 PR, 1 NR | 6-12 months |
| P26-30 | Bomback AS et al; *Am J Nephrol*, 2012 [5] | 05 | Not reported | Three patients received mycophenolate mofetil and 2 patients received cyclosporine plus steroids as first line therapy  Two patients received tacrolimus plus steroids, 2 received cyclophosphamide plus steroids and 1 received mycophenolate mofetil as second line therapy  One patient received rituximab as third line therapy | ACTH | 2 PR, 3 NR | 12-24 months |
| P31-38 | Madan A et al; *BMC Nephrol* 2016 [6] | 08 | Not Reported | Two patients received cyclophosphamide plus steroids, 3 received cyclosporine plus steroids and 3 received tacrolimus plus steroids as first line therapy  One patient received rituximab, 1 received chlorambucil plus steroids and 1 received cyclophosphamide plus steroids as second line therapy | ACTH | 2 CR, 2 PR, 4 NR | 6 months |
| P39-42 | Ramachandran R et al; *Kidney Int* 2016 [7] | 04 | Reported | All 4 patients received tacrolimus plus steroids as first line therapy | cyclophosphamide plus steroids | 1 CR, 2 PR, 1 NR | 06 months |
| P43-62 | Bagachi S et al; *Clin Kidney J* 2018 [8] | 20 | Not reported | Sixteen patients received cyclophosphamide plus steroids and 4 received tacrolimus plus steroids as first line therapy  Ten received tacrolimus plus steroids and 3 received mycophenolate mofetil as second line therapy  One received tacrolimus plus steroids and 2 received mycophenolate mofetil as third line therapy | rituximab | 3 CR, 8 PR, 8 NR | 12 months |
| P63-76 | Ramachandran R et al; *Kidney Int* 2018 [9] | 14 | Reported | Seven received cyclophosphamide plus steroids, 5 received tacrolimus plus steroids and 2 received rituximab as first line therapy | tacrolimus, rituximab, cyclophosphamide plus steroids | 5 CR, 4 PR, 5 NR | 6 months |
| P77-87 | Bagachi S et al; *Kidney Int* 2019 [10] | 11 | Not reported | Nine received cyclophosphamide plus steroids, 1 received rituximab and 1 received tacrolimus plus steroids as first line therapy  Seven received rituximab, 2 received cyclophosphamide plus steroids and 2 received tacrolimus plus steroids as second line therapy  Three received rituximab and 3 received tacrolimus plus steroids as third line therapy | ACTH | 2 CR, 4 PR, 5 NR | Not reported |
| P88-90 | Klomjit N et al; *Am J Kidney Dis* 2020 [11] | 03 | Reported | Two received rituximab and 1 received cyclosporine as first line therapy  Two received rituximab and 1 received cyclophosphamide plus steroids as second line therapy  One received rituximab as third line therapy | obinutuzumab | 2 PR, 1 NR | 9-24 months |
| P91-95 | Sethi S et al; *Kidney Int Rep* 2020 [12] | 05 | Reported | Three received rituximab, 1 received cyclophosphamide plus steroids and 1 received tacrolimus plus steroids as first line therapy  One patient received rituximab as second line therapy | obinutuzumab | 3 CR, 1 PR, 1 NR | 24 months |
| P96 | Geara AS et al; *Glomerular Dis* 2020 [13] | 01 | Reported | Tacrolimus plus steroids was first line therapy and rituximab was second line therapy | bortezomib and dexamethasone | NR | 9 months |
| P97-118 | Ramachandran R et al; *Nephrol Dial Transplant* 2021 [14] | 22 | Reported | Nineteen received cyclophosphamide plus steroids & 3 received tacrolimus plus steroids as first line therapy  One received azathioprine and 1 tacrolimus plus steroids as second line therapy | rituximab | 8 CR, 3 PR, 11 NR | 12-24 months |
| P119-121 | Ramachandran R et al; *Pediatr Nephrol* 2021 [15] | 02 | Reported | One received tacrolimus plus steroids and 1 received cyclophosphamide plus steroids as first line therapy One received azathioprine as second line therapy | rituximab, cyclophosphamide plus steroids | 2 PR, 1 NR | 12 months |
| P122 | Salhi S et al; *Kidney Int* 2021 [16] | 01 | Reported | Rituximab was the first line therapy | bortezomib and dexamethasone | CR | 34 months |
| P123 | Vink CH et al; *Kidney Int* 2022 [17] | 01 | Reported | Mycophenolate mofetil was first line followed by rituximab second line and cyclophosphamide as third line therapy | daratumumab plus rituximab | PR | 9 months |
| P124-125 | Hudson R et al; *BMC Nephrol* 2022 [18] | 02 | Reported | 1st patient was refractory to rituximab and cyclophosphamide.  2^nd^ patient refractory to cyclosporine plus steroids, rituximab and cyclophosphamide | obinutuzumab | 1 PR, 1 NR | 12-15 months |

| \|  \|  \| \| --- \| --- \| |  |
| --- | --- | --- | --- |

ACTH: Adrenocorticotropic Hormone; PR: partial remission; CR: complete remission; NR: No remission; IR: Immunological remission

**Table S3.** Baseline characteristics of included studies

| **Characteristics** | **All included studies** |
| --- | --- |
| Mean age | 43 years |
| Sex (Male : Female)  (reported in 117 patients) | 86:31 |
| Baseline anti-PLA2R antibody level  (reported in 56 patients) | 96.7 RU/ml (IQR 49.8, 222) |
| Baseline 24-hour urine protein  (reported in 108 patients) | 6 grams/day (IQR 4.2, 8.9) |

Abbreviations: PLA2R; phospholipase A2 receptor, IQR; interquartile range

**Table S4.** Summary of treatment received before rescue therapy.

| **Rescue Therapy** | **Number of patients refractory to single treatment strategy** | **Number of patients refractory to two treatment strategy** | **Number of patients refractory to three treatment strategy** |
| --- | --- | --- | --- |
| Rituximab  (n=46) | 30 (23 refractory CYC plus steroids and 7 to tacrolimus) | 13 (9 refractory to CYC plus steroids and tacrolimus, 2 to tacrolimus and MMF, and 2 to CYC plus steroids and azathioprine) | 3 (all three were refractory to MMF, CYC plus steroids and tacrolimus plus steroids) |
| ACTH  (n=34) | 9 (5 refractory to CYC plus steroids and 4 to cyclosporin plus steroids) | 16 (6 refractory to CYC plus steroids and rituximab, 4 to MMF and tacrolimus and, 3 to CYC plus steroids and tacrolimus) | 9 (6 refractory to CYC plus steroids, rituximab, and tacrolimus, 2 to CYC plus steroids, MMF, and tacrolimus and, 1 to rituximab, tacrolimus, and MMF) |
| Obinutuzumab  (n=10) | 6 (5 refractory to rituximab and 1 to tacrolimus) | 1 (rituximab and CYC plus steroids) | 3 (all were refractory to CYC plus steroids, cyclosporin and rituximab) |
| Calcineurin inhibitor plus steroids  (n=13)  (8 tacrolimus and 5 cyclosporin) | 12 (5 refractory to chlorambucil plus steroids and 8 to CYC plus steroids) |  | 1 (refractory to CYC plus steroids, cyclosporin, and MMF) |
| CYC plus steroids  (n=11) | 11 (9 were refractory to tacrolimus and 2 to rituximab) |  |  |
| MMF  (n=8) | 4 (3 refractory to cyclosporin and 1 to CYC plus steroids) | 1 (refractory to CYC plus steroids and cyclosporin) | 3 (all 3 were refractory to chlorambucil plus steroids, CYC plus steroids and cyclosporin) |
| Bortezomib plus dexamethasone  (n=2) | 1 (refractory to rituximab) | 1 (refractory to tacrolimus and rituximab) |  |
| Rituximab plus daratumumab  (n=1) |  | 1 (refractory to CYC plus steroids and rituximab) |  |

**Figure Legends**

**Figure S1:** PRISMA flowchart

**Supplementary References:**

1. DeSanto NG, Capodicasa G, Giordano C. Treatment of idiopathic membranous nephropathy unresponsive to methylprednisolone and chlorambucil with cyclosporin. Am J Nephrol. 1987;7(1):74-6.

2. Miller G, Zimmerman R, 3rd, Radhakrishnan J, Appel G. Use of mycophenolate mofetil in resistant membranous nephropathy. Am J Kidney Dis. 2000;36(2):250-6.

3. Arikan H, Koc M, Cakalagaoglu F, Eren Z, Segal MS, Tuglular S, et al. Tacrolimus rescue therapy in resistant or relapsing cases of primary glomerulonephritis. J Nephrol. 2008;21(5):713-21.

4. Bomback AS, Tumlin JA, Baranski J, Bourdeau JE, Besarab A, Appel AS, et al. Treatment of nephrotic syndrome with adrenocorticotropic hormone (ACTH) gel. Drug Des Devel Ther. 2011;5:147-53.

5. Bomback AS, Canetta PA, Beck LH, Jr., Ayalon R, Radhakrishnan J, Appel GB. Treatment of resistant glomerular diseases with adrenocorticotropic hormone gel: a prospective trial. Am J Nephrol. 2012;36(1):58-67.

6. Madan A, Mijovic-Das S, Stankovic A, Teehan G, Milward AS, Khastgir A. Acthar gel in the treatment of nephrotic syndrome: a multicenter retrospective case series. BMC Nephrol. 2016;17:37.

7. Ramachandran R, Kumar V, Jha V. Cyclical cyclophosphamide and steroids is effective in resistant or relapsing nephrotic syndrome due to M-type phospholipase A2 receptor-related membranous nephropathy after tacrolimus therapy. Kidney Int. 2016;89(6):1401-2.

8. Bagchi S, Subbiah AK, Bhowmik D, Mahajan S, Yadav RK, Kalaivani M, et al. Low-dose Rituximab therapy in resistant idiopathic membranous nephropathy: single-center experience. Clin Kidney J. 2018;11(3):337-41.

9. Ramachandran R, Yadav AK, Sethi J, Gupta KL, Jha V. Antibodies to M-type phospholipase receptor and immunological remission in treatment-resistant and relapsing membranous nephropathy. Kidney Int. 2018;94(4):829-30.

10. Bagchi S, Behera V, Agarwal SK. ACTH (corticotrophin) therapy in resistant primary membranous nephropathy. Kidney Int. 2019;96(1):250-1.

11. Klomjit N, Fervenza FC, Zand L. Successful Treatment of Patients With Refractory PLA(2)R-Associated Membranous Nephropathy With Obinutuzumab: A Report of 3 Cases. Am J Kidney Dis. 2020;76(6):883-8.

12. Sethi S, Kumar S, Lim K, Jordan SC. Obinutuzumab is Effective for the Treatment of Refractory Membranous Nephropathy. Kidney Int Rep. 2020;5(9):1515-8.

13. Geara AS, Bhoj V, Hogan JJ. Bortezomib Treatment for Refractory PLA2R-Positive Membranous Nephropathy. Glomerular Dis. 2021;1(1):40-3.

14. Ramachandran R, Nayak S, Kumar V, Sethi J, Minz R, Kumar V, et al. Rituximab in primary membranous nephropathy: a comparative study of three dosing regimens. Nephrol Dial Transplant. 2021.

15. Ramachandran R, Nayak S, Kumar V, Kumar A, Agrawal N, Bansal R, et al. Primary membranous nephropathy in children and adolescents: a single-centre report from South Asia. Pediatr Nephrol. 2021;36(5):1217-26.

16. Salhi S, Ribes D, Colombat M, Fortenfant F, Faguer S. Bortezomib plus dexamethasone for rituximab-resistant PLA2R(+) membranous nephropathy. Kidney Int. 2021;100(3):708-9.

17. Vink CH, van Cranenbroek B, van der Heijden JW, Koenen H, Wetzels JFM. Daratumumab for multidrug-resistant phospholipase-A2 receptor-related membranous nephropathy. Kidney Int. 2022;101(3):646-7.

18. Hudson R, Rawlings C, Mon SY, Jefferis J, John GT. Treatment resistant M-type phospholipase A2 receptor associated membranous nephropathy responds to obinutuzumab: a report of two cases. BMC Nephrol. 2022;23(1):134.
